# Thirty years of ‘strange bedmates’: The ICPD and the nexus of population control, feminism, and family planning

**DOI:** 10.1080/00324728.2024.2441824

**Published:** 2025-02-04

**Authors:** Leigh Senderowicz, Rishita Nandagiri

**Affiliations:** 1 University of Wisconsin–Madison; 2 Kings College London

**Keywords:** family planning, fertility reduction, ICPD+30, population dynamics, reproductive justice, global health, international development, gender and health, reproductive autonomy, reproductive health

## Abstract

Widely credited with ending population control and ushering in a new era of reproductive rights, the 1994 International Conference on Population and Development (ICPD) Programme of Action also included some important compromises. The commemoration of ICPD+30 presents an opportune moment to reflect critically on those compromises and their implications for family planning programmes in the three decades since. Here, we critically examine how these compromises have enabled population control logics to flourish within global family planning programmes and the ways that neo-Malthusian concerns still motivate contraceptive programming under co-opted feminist rhetoric. We argue that rather than binary stances of ‘pro’ or ‘anti’ contraception, the post-ICPD landscape includes multiple contested positions, including: (1) concern for reproductive rights and autonomy; (2) concern over fertility or population dynamics; and (3) opposition to biomedical contraception and abortion. Setting out the intersecting and diverging tenets of these ideologies, we call for more critical reflection on these tangled histories and engagement with reproductive justice during ICPD+30.

## Introduction

In 1994, delegates to the International Conference on Population and Development (ICPD) in Cairo adopted a Programme of Action (PoA). The Cairo PoA, building on previous work from researchers and advocates, was the first major international policy document to adopt a definition of reproductive rights and to frame women’s empowerment and gender equality as important drivers of economic development (UNFPA 1994). The PoA’s focus on gender and reproductive health represented a substantial shift from the pre-Cairo focus of the population and development community, which had theretofore made population control and fertility reduction their central goals.

In the 30 years since, the Cairo PoA has gained an exalted status in global population, family planning, and reproductive circles. The adoption of the PoA—widely considered the founding document of the contemporary reproductive rights movement—has been described by scholars as a ‘watershed’ event (US Network for Cairo 1994), a ‘touchstone’ for reproductive health (Greer 2006), and a ‘paradigm shift’ for the family planning field (Presser 1997). This ‘Cairo Consensus’ galvanized progress on important issues including maternal health (Thomas et al. 2014), adolescent sexual and reproductive health and rights (Chandra-Mouli et al. 2019), quality of care (World Health Organization 2011), and violence against women (Garcia-Moreno and Amin 2019). Indeed, many advocates believe the PoA was a progressive high point for international reproductive health, integrating feminist approaches and an emphasis on reproductive rights and autonomy (UNFPA 2019), alongside increased funding and visibility for reproductive health (Sippel 2014).

An uncritical veneration of the ICPD, however, risks overlooking the deep compromises that went into the PoA’s crafting, the unease that many feminist activists expressed at the time (Petchesky 1995), and the implications that its concessions, silences, and loose ends have had for reproductive well-being in the years since. At a meeting of the Population Association of America two years after the ICPD, demographers Susan Cotts Watkins and Dennis Hodgson described these compromises and the alliance they helped to forge at Cairo as one between ‘strange bedmates’: feminists on the one hand and population controllers on the other (Hodgson and Watkins 1996). (We note here that the term ‘population controllers’ was widely used and accepted by those motivated by neo-Malthusian concerns to limit population growth in the twentieth century; for more information about the history of the population control movement and the ideological positions behind it, we recommend *Building the Population Bomb* [Merchant 2021]). Hodgson and Watkins noted that these two groups held strikingly different values and equally different motivations for supporting family planning programmes at the ICPD. The population control movement aimed to maintain a focus on reducing population growth among the world’s poor in the Global South and saw family planning as a key means to this end (Hodgson and Watkins 1997; Robinson 2010). Feminists, meanwhile, sought to promote a vision of reproductive health and gender equity, in which all have the autonomy to decide for themselves how many children to have and when to have them (Corrêa et al. 1994a; Sen 1999; Reichenbach and Roseman 2009).

And yet, the common ground that population controllers shared with feminists—the desire to expand access to contraception—united them at Cairo despite their widely disparate starting points. The compromise these groups forged included a concession from population controllers to disavow coercion in their pursuit of lower fertility, while feminists accepted that fertility reduction could still be pursued as a rationale for family planning programmes, as long as all contraceptive use was voluntary (Hodgson and Watkins 1996). Given coercive programmes in the recent past (e.g. forced vasectomies in India, China’s one-child policy), the ‘voluntary’ nature of these programmes was particularly important to both groups (Hendrixson et al. 2014).

In the decades since the ICPD, many of the tense negotiations, compromises, and concessions that went into the Cairo Consensus have fallen away. And in their place, for the most part, is a rosier depiction of the PoA as the feminist document that ended the population control era (Langer 2006). Now, three decades later, as the world reaffirms the commitments of the PoA (Commission on Population and Development 2024) and commemorates the 30th anniversary of the ICPD in 2024 with a series of events under the banner of ‘ICPD+30’, this moment calls for a critical analysis of where the family planning movement has been, how it got here, and how we might reimagine it for feminist futures. It is a particularly urgent moment, given the global backlash against sexual and reproductive health and rights (Sen et al. 2019) and fears of regressing, rather than moving forward, if the ICPD PoA were to be reopened for debate.

In this paper, we argue that the conventional framing of the Cairo Consensus as a feminist high-water mark for global family planning overlooks the fraught compromises and concessions that went into its negotiation and ultimate adoption (Sexton and Nair 2010; Bhatia et al. 2020). Rather, we argue here that the alliance forged at Cairo between those strange bedmates ended up smoothing the multiple fractious positions in population policy and family planning into what appears to be a simple binary: either pro family planning or against it. In this paper we explore the complex ideological positions that intersect, overlap, and diverge across different dimensions of family planning, and we identify the lingering effects of the alliance with population controllers for feminist reproductive health 30 years after the Cairo PoA was adopted.

### Critical feminist approach and roadmap

Rather than systematically reviewing the literature, in this paper we operationalize a critical feminist approach to question the dominant narratives surrounding the ICPD by: (1) synthesizing arguments from an array of disciplines; (2) framing events in their historical context; and (3) proposing a new understanding of the current state of the global family planning field, necessary in the discussions surrounding the 30th anniversary of the ICPD. Our critical feminist lens draws on Connell’s theorization of gender and gendered social embodiment (Connell 2012), as well as Black feminist thinking on reproductive justice (Ross 2017; Luna 2020). Gender is a social phenomenon rather than a static set of categories and is continuously (re)produced through interactions, exchanges, and practices (Connell 1987). In this paper we pay specific attention to gender as a social process to understand how women’s bodies and rights are constructed in deeply gendered ways within the ICPD and the enduring frames this has given rise to.

That access to contraception should be contentious should come as no surprise, as women’s bodies often serve as battlefields in ideological wars. Feminist scholars, such as Frances Mascia-Lees, Nancy Johnson Black, and Barbara Sutton, have explored the idea of women’s bodies as sites of contestation, where larger social anxieties are projected and then policed (Mascia-Lees and Black 1999; Sutton 2010). Dominant conceptions of sex and gender focus primarily on biological roles in human reproduction, but the ways in which these biological roles take on social significance is through what Connell called the process of gendered social embodiment (Connell 2012). This gendered social embodiment is evident in how contraception is experienced and wielded through policy and programming. As Greenhalgh (1990, 1994) has argued, contraceptives can simultaneously empower and disempower women, underscoring how these technologies remain mired in questions of control and power. Riley and Chatterjee (2022), among other critical demographers, have argued that the control of fertility and reproduction (through such technologies) is typically also the control of women. Any attempts, then, to address the health dimensions of reproduction must certainly focus on gender as a key analytic factor but also one that lies within and among other social and political structures (Cornwall 2007).

We use the language of ‘women’ and ‘girls’ in this paper, as much of the reproductive health literature uses a gender binary tied to sex categories and (assumed) biological reproductive capacities. However, we understand these gendered reproductive interventions as affecting trans, non-binary, and gender non-conforming persons as well, and we enthusiastically affirm the need for a more inclusive focus on gender in the reproductive health community. We articulate this more explicitly in our call for a shift to reproductive justice frameworks that move away from an essentializing approach to gender and reproduction.

This paper contains five sections. The first draws on Hodgson and Watkins (1996) to critically examine the alliance of strange bedmates forged at the ICPD, the compromises made there, and how these compromises have shaped the global family planning landscape in the years since. In the second section, we explore how neo-Malthusian family planning programmes have co-opted the rhetoric of women’s health and empowerment since Cairo. In the third section, we complicate the binary conceptualization of family planning ideology forged since Cairo, instead arguing that there are at least three ideological positions within family planning. Identifying the distinct positions—(1) reproductive rights; (2) population and development; and (3) ‘anti-choice’/’pro-life’—we offer a Venn diagram to tease out the overlapping and diverging tenets of each of these positions. In the fourth section, we use a case study of a population, health, and environment (PHE) programme in the Lake Victoria Basin to evidence how these dynamics play out in contemporary global family planning programmes. Finally, we conclude our critical reflections on the ICPD by arguing that the compromises baked into the Cairo PoA are untenable. As we commemorate 30 years of this landmark document, we argue that to achieve progress on reproductive health and reproductive rights, a more critical vision of anti-racist, anti-colonial, feminist reproduct ive justice for global family planning must be advanced.

## Strange bedmates

### Roads leading to Cairo

Debates on the links between population size, population composition, and human well-being have waxed and waned since Malthus’ *Essay on the Principle of Population* (Malthus 1798), but neo-Malthusian concerns about global overpopulation took on a sense of renewed urgency in the middle of the twentieth century. As many former colonies began to gain independence, concern over the links between population size, economic growth, and political stability began to grow in development circles. Coale and Hoover’s influential work, *Population Growth and Economic Development in Low-Income Countries: A Case Study of India’s Prospects* (1958), suggested that population growth would negatively impact economic growth, and their analysis fuelled growing academic and policy concerns that overpopulation would stymie fledgling republics’ attempts to grow their independent economies.

The United Nations (UN) was founded in 1945 and sought to address the issues of global population through a series of world population conferences. Between 1954 and 1994, the UN held five of these decennial conferences. The first World Population Conference was held in Rome and is remembered as a largely academic conference focused on demographic analysis. The second conference, held in Belgrade in 1965, began to move beyond demographic analysis, explicitly discussing the implications for the global development project and indelibly linking these two elements. Conference papers also discussed family planning and other fertility control measures far more than in the 1954 conference (Cox et al. 1966). The third conference, in Bucharest, continued this trend, affirming that ‘population policy must, at the national level, be an integral part of a comprehensive programme of economic and social development’ (United Nations General Assembly 1999, p. 40). By 1976, every country in the UN had proclaimed an official stance on fertility, whether a policy on lowering, maintaining, or increasing it. Of 150 countries, 95 governments directly supported family planning programmes, 17 provided indirect support, and 10 sought to limit family planning programmes, while the other 28 countries had no formal family planning policies (United Nations Population Division, n.d.). All 55 countries who viewed their fertility as ‘too high’ were classified by the UN as ‘less developed’.

In the meantime, overpopulation became a central motivation for bilateral development funding in the immediate post-colonial era. The United States Agency for International Development (USAID) was founded in 1961, and family planning research was one of the very first activities it supported in that same year (USAID 2024a). By 1969, US President Richard Nixon had deemed population growth ‘one of the most serious challenges to human destiny’, and the Office of Population had been established within USAID to coordinate fertility reduction and family planning activities as a way to address population growth (USAID 2024a). Cold War tensions shaped development assistance and aid flows, with funding for family planning programmes expanding as a result. In addition to global tensions, USAID’s family planning funding was shaped by United States (US) domestic politics and ideologies. For example, in 1973 conservative US senator Jesse Helms sponsored what came to be known as the Helms Amendment, a law that limits the use of US foreign assistance for supporting abortion care.

The fourth conference, the 1984 International Conference on Population, is perhaps best remembered for the implementation of the Mexico City Policy (also known as the Global Gag Rule), mandating that recipients of US funding not engage in any abortion-related activities, even with funding from other sources (Cohen 2001). The US’ primary ally at this conference was the Holy See, reflecting the conservative ideological position of the administration in power. Conservative politics linked opposition to abortion with opposition to forced sterilization, and US development policy gradually extended the opposition to forced sterilization into principles of voluntarism that covered reversible methods of contraception as well (Kaiser Family Foundation 2024). Notably, opposition to coercive family planning stemmed initially from conservative politics, before they were explicitly associated with a feminist rationale. By the time of the next conference, proponents of this conservative anti-contraception ideology were no longer in power in the US, having been replaced by a more liberal administration that was broadly supportive of reproductive rights. These geopolitical, economic, and ideological development frames characterized much of the discussion surrounding population and family planning in the run-up to the 1994 ICPD in Cairo.

### Cairo’s strange bedmates and an ‘almost feminist vision’ of sexual and reproductive health and rights

The Cairo conference is widely regarded as a watershed moment in women’s health and rights and is routinely described as a ‘milestone’ and a ‘turning point’ for family planning (Singh 2013; Ashford 2014; International Planned Parenthood Federation 2014). In 2006, reproductive health scholar Ana Langer wrote that:

The Cairo conference put the ideas of comprehensive sexual and reproductive health and rights, choice, women’s empowerment, a life-cycle approach, and gender equity at the centre of the international agenda, and signalled the end of the so-called population era. Instead of pursuing demographic targets via family-planning programmes, the goals of the ICPD Programme of Action (signed by 179 countries) were to achieve universal access to safe, affordable, and effective reproductive health care and services, including those for young people, and promoted a gender perspective. (Langer 2006, p. 1552)

The shift from the so-called ‘population era’ to such an ambitious feminist agenda has been much analysed and debated, with many scholars crediting the shift to an active and well-organized feminist presence at the ICPD (Friedman 2003). Although some have claimed that the feminist presence at the ICPD was dominated by feminists from the Global North, it is important to note that feminists from the Global South significantly shaped and contributed to the agenda, demanding an explicit recognition of entrenched social, economic, political, and structural injustices as interlinked with sexual and reproductive health (Corrêa et al. 2005; Sen 2019).

Feminist gains at Cairo were not achieved on principles alone. Demographer Rachel Robinson described how and why feminists forged a strategic alliance there with the neo-Malthusian ‘population establishment’:

Both neo-Malthusians and feminists had something to gain from coming together at Cairo. By banding with feminists, neo-Malthusians gained the advantage of a frame for their ultimate goal (reduced fertility) expressed in the more politically correct form of women’s rights and wellbeing. By allying with neo-Malthusians, feminists maintained a tie to the population establishment. Both groups’ support for abortion, which constructed the Vatican as a shared enemy, helped facilitate the partnership. (Robinson 2010, p. 6)

The PoA that emerged from this alliance made an important break from past conference documents, calling for a human rights approach to family planning rather than a focus on demographic outcomes alone. And yet, contrary to popular belief, neo-Malthusian values still abounded in the Cairo PoA, which retained an explicit focus on population size and its effect on sustainable development. Section 3.14, for example, states that slowing population growth increases:

[…] countries’ ability to attack poverty, protect and repair the environment, and build the base for future sustainable development. Even the difference of a single decade in the transition to stabilization levels of fertility can have a considerable positive impact on quality of life. (UNFPA 1994)

This continued linking between fertility and development in the PoA, however, was insufficient for many scholars, who argued that the broader focus on reproductive rights took an important spotlight away from family planning more specifically (Cleland 1996). Others suggested that the expanded approach of ‘reproductive health’ may have downgraded family planning programmes (Gillespie 2004), risked programmes being pulled in multiple directions (Jain 1995), and/or reflected a ‘misperception that the population crisis is over’ (Langer 2006, p. 1553), all of which, they argued, diminished the funding and focus of family planning programmes. Despite these critiques however, the general consensus around the ICPD PoA has been one of veneration for its deft interweaving of reproductive rights and fertility concerns into a common agenda.

However, the veneration of the Cairo Consensus today as a watershed for reproductive rights overlooks much of the compromise that went into the document’s ratification. While the PoA has been praised for being shaped largely by a feminist ethos (Corrêa et al. 2005; Sen 2019) and centring women’s voices along with demographic rationales for the first time (Balakrishnan 1996; Klugman 1996), mostly forgotten now are the many feminist critiques of the PoA on key issues and, indeed, how much debate and contestation there was within feminist circles at the time (Cornwall et al. 2008; Sexton and Nair 2010; Bhatia et al. 2020). Feminist voices—including many of those who helped shape the PoA—considered the result to be a ‘compromise consensus’ (Berer 2009; Sippel 2014), which ceded many key feminist principles (e.g. an opposition to neoliberalism) to political and strategic interests and fell well short of its intended feminist visions. Rosalind Petchesky described these compromises as ‘standing on a fault line’, where the ICPD enshrined an ‘almost feminist vision’ of gender equality and reproductive rights, while still retaining an uncritical and ‘mainstream model of development under which that vision cannot possibly be realized’ (Petchesky 1995, p. 152). Other critics, such as Betsy Hartmann warned that without careful attention to dismantling structural inequalities, reproductive health would remain an unfulfilled feminist goal, and the Cairo compromises would instead function as a palatable cover for continuing neo-Malthusian aims (Hartmann 1987, 1997a, 1997b).

Among the most consequential feminist compromises made in the ICPD PoA were silences on the environment and racism (Halfon 2007; Sasser 2017). Corrêa et al. (1994b) located the ICPD’s silence on the environment within a broader shift, exemplified by Ehrlich’s Population Bomb (1971), that linked population control with environmental balance. They highlighted how the ICPD’s compromise was not unrelated to the alliance forged between the ‘population establishment’ and environmentalists at the 1992 UN Conference on Environment and Development just two years prior. Balakrishnan noted that the ICPD failed to reframe the causes of environmental degradation as related to unchecked consumption or inequitable distribution of resources, leaving assumptions of the links between population growth, economic well-being, and sustainable development untroubled (Balakrishnan 1996).

Petchesky also highlighted the ICPD PoA’s silence on racism and colonialism as structural forces that dramatically shape the conditions under which people access reproductive healthcare (Petchesky 1995). She argued that this silence was not just a missed opportunity to grapple with entrenched inequalities but a direct reflection (and perpetuation) of the racism and eugenicist doctrines that had underpinned population policies and programmes throughout the twentieth century (see e.g. Greenhalgh 1986; Palen 1986; Grimes 1998; Reilly 2015; Robinson 2015; Dyck and Lux 2016). These silences around racism and the environment, Petchesky (1995) suggested, were reflective largely of the divisions between the Global North and the Global South, the latter including minoritized populations within the Global North. White Northern feminists failed to grapple with intersecting axes of marginalization, resulting in a PoA that re-etched inequitable power dynamics rather than confronted them. Indeed, in the lead up to the ICPD, some Southern and critical feminists warned that efforts to reform population programmes into rights-based programmes, given their origins in racist and eugenicist beliefs, were an inherent contradiction (Petchesky 1995; Nair et al. 2005). They cautioned that any efforts to ally feminist aims with neo-Malthusian ones would see the former co-opted in service of the latter.

## Co-opted rhetoric and the blurring of contemporary ‘feminist fault lines’

Feminist concerns about the compromises baked into the ICPD PoA ended up being well founded, as many of the consequences and forms of co-optation they predicted have indeed come to pass. In the post-Cairo era, the clear distinctions that previously separated feminist organizations from population control organizations have blurred considerably, as the language of reproductive health and rights has been used to motivate the vast majority of global family planning programmes.

Reviewing the language of current global family planning organizations—both those with feminist origins and those with population control origins—highlights how these strange bedmates have evolved to resemble one another so closely in the intervening years. In Table 1 we present relevant excerpts from the strategic documents and mission statements of influential sexual and reproductive health and rights (SRHR) and family planning organizations, including those with their origins in feminist advocacy as well as those with origins in the population control movement. We explore these organizations, given their prominent role in the ICPD meetings and in formulating the PoA. These organizations continue to play key roles in SRHR and family planning agenda-setting today (Brueggemann 1997; Catley-Carlson 1997; Claeys 2010; Germain 2018). What we find is that despite their disparate starting points, these two camps might not seem like such strange bedmates any more. The ‘common ground’ cause of expanding access to contraception has so thoroughly united these groups that they cannot be easily disentangled any more. We find that the rhetoric used to motivate family planning programmes is similarly rights-based and equity-focused across this range of organizations, regardless of their origins or historical mandate. With recurrent themes such as women’s empowerment, women’s health, and gender equity, it has become difficult to parse which organizations are concerned primarily with feminist aims, which are more concerned with demographic outcomes, and if a distinction is still possible.

The increased reliance on rights-based and person-centred language in family planning would be apt if it reflected a true shift away from neo-Malthusian ideology and a thorough dismantling of population control structures from within the global family planning establishment. In many cases, however, the dramatic *rhetorical* shift of family planning away from population control in the post-ICPD world has not been accompanied by a commensurate *substantive* shift away from fertility reduction activities and programmes (Senderowicz 2020; Nandagiri 2021). Rather, leaders in the global family planning community (including from the Global South) have blended rights-based rationales with instrumentalist rationales to motivate family planning programmes and advocacy (Senderowicz and Valley 2023). Yet reducing fertility, predominantly among poor women of colour living in ‘overpopulated’ parts of Africa and South Asia, remains a primary goal of many family planning projects. But with the term ‘population control’ essentially verboten in the post-Cairo world, these initiatives now bask instead under the politically correct buzzwords of ‘gender empowerment’ and ‘reproductive health’.

One of the key euphemisms for signalling fertility reduction in a way compatible with appropriated feminist buzzwords around health and empowerment has been the term ‘population dynamics’. Including demographic concerns around population growth, age structures, urbanization, and migration, population dynamics have increasingly been articulated as part of a ‘common agenda’ of global development and reproductive rights (Newman et al. 2014), where family planning acts as a climate mitigation strategy while also delivering better health outcomes, empowerment, and poverty reduction (Stephenson et al. 2010). The USAID website, for example, explains that benefits of family planning include ‘mitigat[ing] the impact of population dynamics on natural resources and state stability’ (USAID 2024b). They expand on the link between family planning and climate change, stating,

Incorporating access to sexual and reproductive health services, including voluntary contraception, among a set of holistic interventions can mitigate these [climate] risks, enabling girls and women to delay and plan their pregnancies and better protect their health and that of their children—which, in turn, helps build resilience at the individual, household and community level. (USAID 2024c)

Many contemporary family planning initiatives work with major funders, non-governmental organizations, and private companies to set influential SRHR norms and policies. Family Planning 2020 (FP2020; now FP2030), for example, is funded in large part by the Bill & Melinda Gates Foundation and has been a leading player in setting the agenda for the global family planning community since 2012. FP2020 played an important role in reintroducing and re-legitimizing quantitative targets for contraceptive uptake (Hendrixson 2019), while also taking care to disavow population control explicitly in its advocacy materials. FP2020 has worked to frame its pursuit of contraceptive uptake in the context of finding a common language that marries family planning with population dynamics and sustainability in a rights-based frame. An FP2020 workshop in 2015, for example, urged the SRHR community ‘to take real leadership on population dynamics while remaining true to our commitment to rights, empowerment, and women’s and girls’ autonomy and agency in FP decisions’ (FP2020 2019, p. 23). As applied family planning scholarship has shown, there is a lag in moving away from supply–demand approaches and towards centring agency and reproductive power (Galavotti and Gullo 2022; Marston and Tabot 2023). Indeed, while the overwhelming majority of FP2020 advocacy and policy work was framed using this language of rights, empowerment, and autonomy, the central pillar of the FP2020 initiative was to add ‘120 million additional users of modern contraceptives by 2020 in the world’s poorest countries’ (Brown et al. 2014, p. 73): a quantitative uptake target reminiscent of the pre-Cairo quotas for new family planning ‘acceptors’ (Lapham and Mauldin 1972; Phillips 1978).

The co-optation of feminist reproductive health rhetoric is emblematic of the shifts around gender empowerment more broadly. Cornwall has argued that once-contested development buzzwords such as ‘empowerment’ have been flattened into vague euphemisms that can ‘embrace a multitude of possible meanings’ (2010, p. 2). This ambiguity enables endorsement by a range of powerful actors but also makes these concepts vulnerable to misappropriation and to a more general watering down (Batliwala 2010). Even where feminist and equity-related principles are integrated into family planning frameworks, they are often framed in service of demographic aims. Judith Bruce, for example, underscored that ‘the availability of services of reasonable quality will be of humane value to the prospective clients and, over time, should assist the achievement of national demographic goals’, demonstrating how she viewed ‘contraceptive use: acceptance [and] continuation’ as primary goals, even in this foundational treatise on quality (Bruce 1990, p. 62). More recently, Hardee, Kumar, et al. (2014) offered a similar argument in their conceptual framework on ‘voluntary, human rights-based family planning’, where they highlighted a decrease in total fertility as a key outcome of their proposed approach. In response, feminist reproductive health scholars have argued that high standards of care and a focus on rights must be understood as essential in and of themselves, irrespective of their ultimate impact on contraceptive behaviours and fertility outcomes (Corrêa et al. 2005; Reichenbach and Roseman 2011; Senderowicz 2020; Senderowicz et al. 2021).

Many in the contemporary family planning community have linked voluntary family planning with a range of benefits for sustainable development, framing it as mutually beneficial for both women’s empowerment and those other goals, with coercion largely a problem of the past (Foley 2007). Evidence shows, however, that as long as family planning programmes are motivated by these kinds of instrumentalist goals, there is a real risk of reproductive rights and agency being subordinated to their pursuit. Evidence from an anonymized country taking part in the FP2020 initiative, for example, linked the pursuit of additional user targets with pressure to adopt contraceptive methods and even to outright coercion (Senderowicz 2019). Studies from other FP2020 countries have found that providers may be reluctant to remove contraceptive implants on request or otherwise help users to discontinue a method they no longer wish to use (Callahan et al. 2020; Yirgu et al. 2020; Britton et al. 2021). This empirical evidence, among a range of other historical, legal, and journalistic sources, leads us to conclude that although feminist rhetoric has been fully integrated into mainstream global family planning programmes, the feminist ideals, values, and practices that this language originally drew from are largely absent from them.

## Expanding binary conceptualizations of family planning ideology

Tracing how feminists and population controllers came to be strange bedmates highlights how, besides a shared belief that contraception should be available to women, there was initially little common ground between the two groups. Yet over the intervening decades, the complexity of their ideological positions has been smoothed over and presented to the world simply as a ‘pro-family-planning’ stance, in contrast to those opposed to the promotion of biomedical methods of contraception. The tension inherent in this alliance is seldom acknowledged today by its members, although it has been implicitly recognized through the family planning community’s focus on ‘quality of care’, ‘provider bias’, and ‘voluntarism’. To the extent that mainstream family planning advocates have sought to address coercion head-on, they have framed it largely as something that happened in the past (Gold 2014; Hardee, Harris, et al. 2014) or something that is rare and limited to a few bad apples (Bongaarts and Sinding 2009). Combining this framing with rights-based arguments (i.e. contraception is good because control of one’s own reproduction is a human right) and with instrumentalist arguments (i.e. contraception is good because it contributes to goals such as poverty reduction or environmental protection), the pro-family-planning alliance has developed a strong narrative that frames contraception as an unambiguous good for all (Senderowicz and Valley 2023). This framing has left little room for critiques of neo-Malthusian approaches to family planning to be understood in good faith and has instead framed critiques of mainstream global family planning programmes as stemming from a bad-faith opposition to family planning writ large.

While the post-Cairo conceptualizations of family planning ideology are framed as a binary of either pro or anti global family planning, this understanding papers over important differences held by those who support family planning (Halfon 2006). Indeed, there are at least three main ideological positions within this contested sphere. The positions include those: (1) concerned with reproductive rights, autonomy, and freedom; (2) those concerned with the effects of fertility and population size on global development; and (3) those opposed to medicalized contraception and abortion (commonly referred to as a pro-life or anti-choice position). These three positions shift, overlap, intersect, and diverge around different tenets that do not fit neatly into the binary conceptualization of family planning ideology that has been so dominant in the global health arena over the last quarter century.

### Reproductive rights

The group(s) concerned with reproductive rights could be understood to correspond roughly with the feminists that Hodgson and Watkins identified at the ICPD. Here, we use this term to mean those who believe that access to contraception is not a means to an end (e.g. protecting the environment, encouraging women’s labour force participation, reducing poverty, etc.) but, rather, an end in itself. This group’s focus is on ensuring that gendered bodies have full control over their own reproduction, as well as ensuring that they are in a suitable position to be able to make these decisions about how they want their families to look. In our conceptualization of this ideological position, supporters of contraception for reasons of reproductive rights back access to a full range of methods and believe that people capable of getting pregnant can best make their own choices about which kinds of methods to use. These might include fertility-awareness-based methods (FABMs, also known as ‘traditional’ or ‘natural’ methods) as well as biomedical (‘modern’) methods of contraception. Support for comprehensive abortion care as part of the spectrum of sexual and reproductive healthcare is integral to this ideological position, as this community believes that bodily autonomy is at the very heart of reproductive rights rather than something that can be siloed off.

### Population and development

Those concerned with the effects of fertility and population will overlap with the reproductive rights group in their shared support for biomedical methods of contraception and for wide expansion of access to family planning programmes. This group sees low total fertility and high contraceptive prevalence as the ultimate goals of family planning programmes, as fertility reduction is seen to be important to achieving other social, economic, ecological, or development goals (Ezeh et al. 2012; Fabic et al. 2015; Starbird et al. 2016). The population and development group is focused on the efficacy of contraceptive methods, placing a strong emphasis on long-acting methods of contraception, often at the expense of FABMs and less effective, shorter-acting methods. Due to their belief that family planning has instrumental benefits that go beyond the autonomy of the user, this group also believes in the importance of ‘demand creation’ for contraception in women who do not currently wish to use it. This group’s demand creation activities can take a number of forms but often include social marketing and behavioural change communications (Mwaikambo et al. 2011). Although many of the people working within this group may support abortion rights individually, they are often not permitted to work on this as a matter of policy. Thus, a strong stance on abortion advocacy is often relinquished by this group for the sake of political expediency (Hempel 1996; Seltzer 2002; Hodgson 2009).

### Pro-life/anti-choice

According to conventional post-ICPD wisdom, these first two groups are allied with each other in opposition to the third group: those opposed to the use of biomedical contraception and abortion. However, we posit here that rather than being simply the polar opposite of either the reproductive rights and/or population and development camps, the third group displays unexpected areas of overlap with both these groups, in addition to the areas of strong disagreement. This anti-choice group is often—but not always—religiously motivated. While many adherents of this anti-choice ideology claim that religious doctrine is at the root of their beliefs, others may be secular and instead be inspired by a New Age understanding of ‘wellness’ that emphasizes natural methods and disdains the use of exogenous hormones in many biomedical methods of contraception.

The anti-choice contingent overlaps with the reproductive rights group in their belief that FABMs are a good choice for people who want to use them. Along with the population and development group, the anti-choice group shares a belief that certain methods of contraception are better for users than other methods. In the case of the anti-choice group, they may oppose barrier, surgical, hormonal, and other biomedical methods of contraception, believing so-called natural methods (FABMs) to be superior. This is essentially the mirror image of the population and development group’s belief that *only* biomedical methods should be used. Both of these positions come from a paternalistic notion that users should benefit from only a small subset of contraceptive options, rather than a broad contraceptive method mix. In contrast to the reproductive rights group, this anti-choice group is staunchly opposed to abortion care and has made fighting abortion rights one of its primary agenda items.

### Points of overlap and divergence between ideological camps

**Figure 1:**
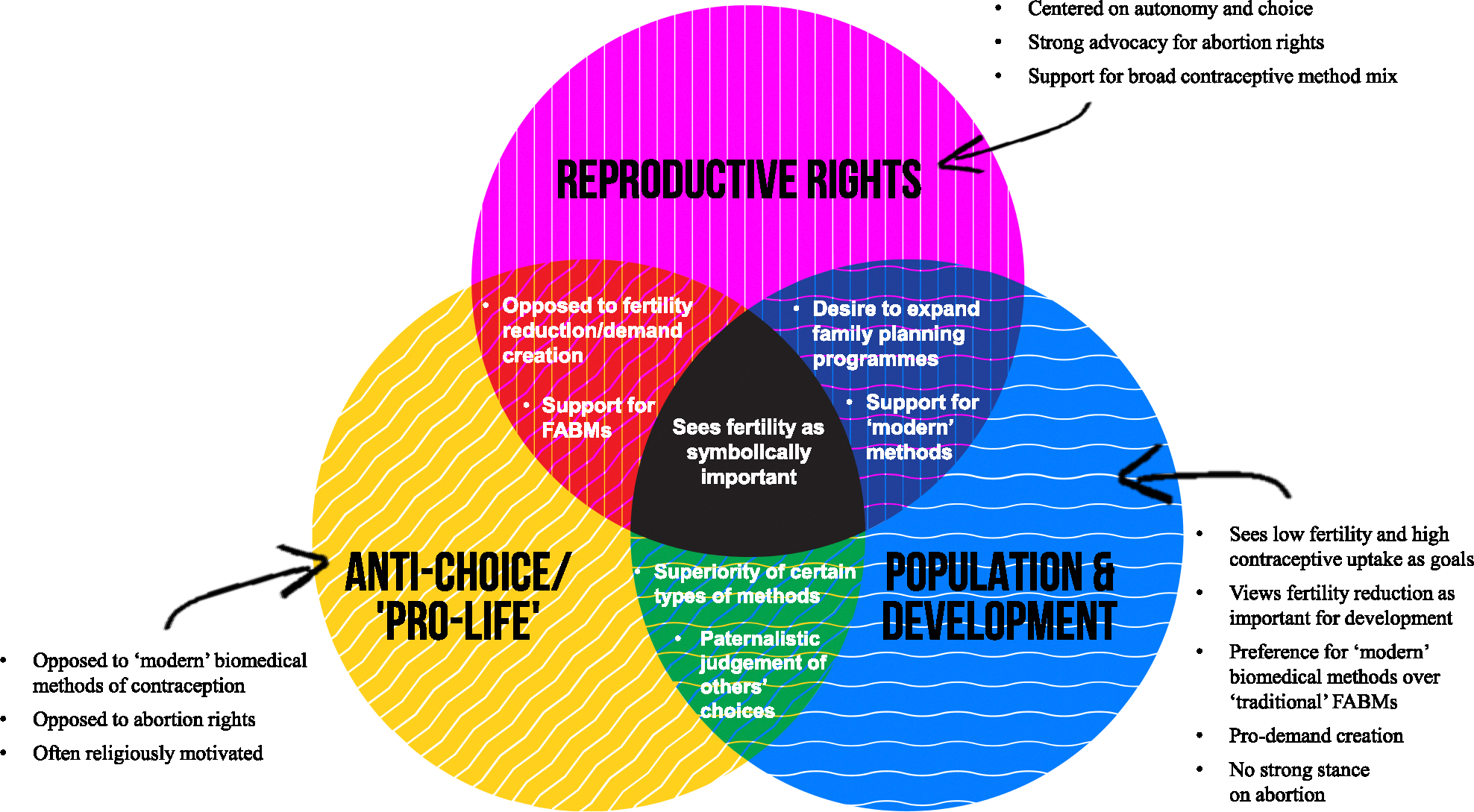
A Venn diagram of overlapping beliefs by ideological camps

Figure 1 sets out the points of overlap and divergence between these groups. All three groups agree on the symbolic importance of a woman’s reproductive capacities. For the reproductive rights group, fertility is symbolic of gender equality and of autonomy, as the ability to choose abortion, childbirth, contraception, or conception are of special importance to the ability to make life choices. Fertility is symbolic for the development group, too, with low fertility both representing and enabling modernity and progress. For the anti-choice group, fertility is symbolic of motherhood and adherence to traditional gender roles.

As the Venn diagram in Figure 1 shows, there are indeed meaningful points of agreement between the population and development group and the reproductive rights group, serving as the keystone on which the strange bedmates’ alliance has relied. However, this shared belief in expanding access to contraception is not sufficient to glaze over the profound differences that divide these two groups, even though the lines between them have blurred since the ICPD. The population and development camp, for example, shares with the anti-choice camp beliefs in limiting the types of choices that women are able to make about their bodies and their fertility and in promoting certain types of contraceptive methods and fertility choices over others.

The dominant view within global SRHR has traditionally seen the anti-choice movement as diametrically opposed to the pro-family-planning alliance. Our argument here challenges this view, suggesting that they are not in diametric opposition to each other but have more complicated and interesting overlaps, strategic alliances, and important points of discord, not just ‘compromises’. The point of this argument for proponents of reproductive rights is not to embrace the anti-choice movement (and their attempts to restrict reproductive freedom) more warmly but, rather, to see that many of the same principles so abhorred in the anti-choice movement can also be found among supposed allies in the fertility and development camp.

In recent years, even though neo-Malthusian ideology has continued to dominate the fertility and development literature and activism in the Global South, a resurgent pronatalist ideology has emerged in the Global North (Spears 2023). Here, concerns about ageing populations and declining fertility rates (‘depopulation’) have begun to dominate research and policy discussions, including among many in the fertility and development camp (Geruso and Spears 2023). This pronatalist ideology can work on two fronts: (1) aligning with the anti-choice group in discouraging contraceptive use and restricting abortion to tackle low fertility rates; and (2) encouraging increased childbearing. For some pronatalists, this ideology is rooted in racist and far-right conspiracies (e.g. the ‘great replacement’; Talbot 2024).

Although contrasting in many ways, pronatalist and anti-natalist positions are grounded in similar concerns: that women’s ‘suboptimal’ childbearing—either too few children or too many—will have deleterious impacts on economic growth or other social goals. This leads to the conclusion from both camps that fertility rates must be modified—either upwards or downwards—to engineer optimal societal outcomes. This instrumentalization of women’s bodies is discussed at great length in other work (Senderowicz and Valley 2023), but we highlight here the ways in which this instrumentalization of women’s reproductive capacities and control over women’s bodies are a key feature of both the pro- and anti-natalist ideologies, underscoring how these opposing beliefs are essentially two sides of the same coin.

## Population, health, and environment: A case study

One example of the how neo-Malthusian ideology has been recast with co-opted progressive language and a ‘common agenda’ framing that includes both rights-based and instrumentalist rationales is the PHE approach used in low-income countries, including the Philippines, Nepal, Madagascar, and Ethiopia (Gonsalves et al. 2015; Hoke et al. 2015; Robson et al. 2017; Sasser 2017; Baker-Médard and Sasser 2020). PHE links family planning and reproductive health services with environmental conservation efforts in communities that are often on the frontline of the climate emergency. These programmes include efforts to ‘educate communities about the relationship between rapid population growth and environmental degradation’, and ‘by facilitating access to family planning services and publicizing their role in preventing unplanned pregnancies, environmental programmes can offer a practical, immediate action that contributes to future conservation of natural resources’ (Hoke et al. 2015, p. 43).

The Health of People and Environment in the Lake Victoria Basin (HoPE-LVB) project, implemented in Uganda and Kenya in 2011–17, is emblematic of a PHE approach. Pathfinder International was the lead agency, working closely with a number of local and national organizations, including conservationists and local governments. Activities were funded by the MacArthur Foundation, the Packard Foundation, and USAID, among others. Implemented over two phases, the project used a two-pronged intervention consisting of: (1) a fisheries management programme; and (2) a family planning programme. A briefing document about the project’s Beach Management Units, titled ‘Bringing Fishing and Planning for Families Together in the Basin’, summarized the problem and proposed solution:

Over time, unsustainable fishing and farming practices, as well as increased demand for resources from rapidly growing population, has overwhelmed fisheries that have traditionally supported the basin. In the last few decades, activities in the basin have increasingly created stress, resulting in the environmental instability of the lake. […] A new approach to conservation in the basin—to save families as well as the fish and their habitats —is the Health of People and Environment in the Lake Victoria Basin (HoPE-LVB) project. HoPE-LVB seeks to reduce threats to biodiversity conservation and ecosystem degradation in the LVB while simultaneously increasing access to family planning and reproductive health services, in order to improve maternal and child health in project communities in Uganda and Kenya. By integrating the delivery of reproductive health, livelihood, and conservation education and services in these communities, HoPE-LVB is improving reproductive health and natural resource management in the basin more successfully than programs focused exclusively on reproductive health or the environment. (Pathfinder International 2016, p. 1)

On the face of it, this intervention married two favourite progressive causes—women’s rights and environmental protection—for a win–win solution to a crucial global health problem. On closer examination, however, the programme actually used a regressive neo-Malthusian lens to understand the nature of the problem and then built a solution that targeted population control measures on the bodies of Black women living in poverty in the Global South.

This programme chose to understand the fisheries challenge in the Lake Victoria Basin as one of overpopulation as a way to make sense of why the supply of fish in the lake could not meet the demand for food from the people living around the lake. What this programme did not include, however, was a more holistic exploration of the role of extractive global commercial interests as root causes of the environmental degradation and poverty in Lake Victoria Basin communities. For example, nowhere in the HoPE-LVB programme was there an attempt to account for the fact that the ‘use of fertilizers and animal manure’ had ‘increased tremendously, causing accelerated eutrophication of Lake Victoria waters’ (Ntiba et al. 2001, p. 1). The programme did not mention Lake Victoria Basin Commission’s basin-wide strategy (2012), which linked the degradation of fisheries in the lake to pollution, such as fuel and oil spills or untreated liquid waste from mining and other industrial activities. The rapid increase of water hyacinth—an invasive species that badly affects fishing and water quality—was similarly not mentioned (Hammond and Xie 2020).

Rather than seeking to engage with the ways that extractive industries and invasive species were reducing the ability of the lake to sustain healthy fisheries, the HoPE-LVB project instead relied on neo-Malthusian arguments about population growth outstripping food supply. By targeting family planning and reduced fertility as the solution, it implicitly cast the reproductive bodies of the people who lived there as the cause of the problem. Such efforts individualize responsibility for tackling the problem of unsustainable practices but frame the intervention—use of family planning—as both empowering and a reflection of empowerment. Thus, the language of justice and empowerment comes to be instrumentalized to promote fertility reduction (Sasser 2018), and women’s health and well-being become cast as a smart investment in sustainability. Similar PHE programmes in other contexts, such as Ethiopia, have been lauded for increasing family planning uptake. Such ‘common agenda’ programmes are a microcosm of the larger mainstream family planning space: the commitment to access to contraception remains tied to the logics of fertility control as a means to foster development.

That people who are being targeted by these family planning programmes are poor Black women living in the Global South is central rather than incidental to the ideological underpinnings of PHE programmes and of global family planning more broadly (Kuumba 1999; Sasser 2014). Indeed, much of what we observe now reflects the concerns raised by critical feminist groups at and after the ICPD: that the silences around race and racism, the uncritical and mainstream approach to ‘empowerment’ without tackling the conditions that give rise to disempowerment, and the absence of challenging the frames of fertility control or population stabilization would all result in the instrumentalization of women under the guise of achieving rights. By targeting fertility as a solution to the fisheries problem in Lake Victoria Basin instead of environmental pollutants from extractive global industries, the programme implicitly blamed women’s bodies and behaviours for poverty, food insecurity, and biological degradation, without any mention of structural factors, shrouding these race-, gender-, and class-based programmes in co-opted language about women’s empowerment and health.

## An anti-racist, anti-colonial, feminist vision of reproductive justice

Tracing the compromises that shaped the PoA and the co-optation of critical concepts into buzzwords is a first step in interrogating how the ICPD and its ideals have evolved within organizations and institutions, contributing to new discourses and rhetorics in broader family planning and reproductive health efforts. We are currently seeing increasing attacks on abortion and reproductive health (including draconian pregnancy surveillance laws in Poland (Gera 2022) and the increasing criminalization of abortion in the US (Hoffman 2022)), growing transphobia, and increased attacks against gender-affirming care (Allegrozzi 2021; Martin et al. 2021), along with transnational efforts challenging evidence-based SRHR efforts (including the Geneva Consensus Declaration [Gilby et al. 2021] and increased opposition at the World Health Assembly to language on sexual health [Heilprin and Fletcher 2022]). At this moment of crisis, it is essential to reflect on the continued utility of past compromises.

The concept of reproductive justice is one that evolved in the US in the mid-1990s, as Black American feminists returned from the ICPD and sought to integrate the feminist human rights frameworks into the US movement for reproductive freedom. Whereas the mainstream feminist organizations in the US were led mostly by white, middle-class women focused on removing legal barriers to abortion and access to contraception, women of colour saw legal barriers as only part of the problem. For poor women, women of colour, institutionalized women, and other marginalized groups, including trans, non-binary, and gender non-conforming persons for whom the legacy of eugenics was (and is) very much present, the right to be able to have the children and create the families they wanted was just as much in doubt as their ability to prevent the pregnancies they did not want (Radi 2020). The reproductive justice framework acknowledges the ways in which racism, homophobia, transphobia, poverty, and other forms of structural oppression de facto prevent many from being able to exercise their autonomy, even in contexts free of de jure (legal) restrictions to reproductive health services (Luna 2009; Ross 2017), deepening understandings of and approaches to reproductive self-determination.

Although developed in the US context, reproductive justice critiques and frameworks are useful in the global health and development context as well. As poor women of colour living in poor countries see their fertility implicitly blamed for everything, from global warming to food scarcity and their own poverty, and see their reproductive choices constrained by the structural violence that operates on them (Maternowska 2006; Nandagiri et al. 2020), it becomes clear that focusing simply on the choice to use contraception is fundamentally missing the point. The women in the Lake Victoria Basin who saw industrial agricultural run-off and mining-related contamination kill off a key food source were not met with feminist solidarity to help them stand up to the transnational business interests that were sullying their environment. Instead, they were met with measures that sought to reduce their fertility while using the language of ‘empowerment’. In the international context, reproductive justice interweaves reproductive health themes (e.g. access to safe abortion, to a broad range of contraceptive methods, to safe motherhood) with efforts to address the structural and historical factors that keep women in poverty, perpetuate racism and coloniality, and contribute to gender oppression, including by ‘bedmates’ in the development community (Chiweshe et al. 2017; Wilson 2018; Gondouin et al. 2020; Grabe et al. 2020; Macleod et al. 2022).

Informed by the neo-Malthusian framing that population growth is an important contributor to climate catastrophe, many still harbour the view that environmental concerns sit in a fundamental tension with an understanding of reproductive rights that allows for and even encourages people to choose large families. Neo-Malthusian scholars have argued that fertility reduction is necessary to protect biodiversity and alleviate climate change and also that allowing people to choose large families is fundamentally incompatible with long-term environmental sustainability (Ehrlich 1968; Potts et al. 2011; Mohan and Shellard 2014). These arguments have been repeatedly debunked on their scientific merits, with climate scientists, for example, showing that runaway consumption among the world’s wealthiest, rather than the fertility of the world’s poorest, is the overwhelming cause of CO_2_ emissions (Davis and Caldeira 2010; Kenner 2019). Nevertheless, environmental concerns are often used as a justification for continued opposition to high fertility and for continued calls to renew the types of compromise made in the ICPD PoA (Das Gupta et al. 2011; Newman et al. 2014; Mayhew et al. 2020).

The expansiveness of the reproductive justice framework and its intrinsic connections with other social justice movements helps to confront and disrupt the neo-Malthusian thinking that frames reproductive freedom and environmental protection as opposing struggles (Di Chiro 2008; Gaard 2010; Appleton 2022; Sasser 2024). As the HoPE-LVB example shows, the same marginalized groups who are most likely to be targeted for fertility reduction are also those most likely to incur the direct consequences of environmental degradation. Thus, any solutions to these problems must address their interconnected root causes and prioritize the sovereignty and self-determination of the most marginalized. Reproductive justice, then, *is* environmental justice and vice versa (De Onís 2019; Liddell and Kington 2021; Goldstein 2022; Sasser 2024).

## Conclusion

In 1997, Hodgson and Watkins speculated that the alliance between feminists and neo-Malthusians forged at Cairo was so fragile—’based on a fabricated unity of interests that neither movement finds convincing’—that it was ‘unlikely to endure’ into the twenty-first century (Hodgson and Watkins 1997, p. 510). Yet, three decades on, this alliance has calcified and been naturalized to the point where a reproductive health movement that also seeks to influence fertility trends seems self-evident and unassailable. But Hodgson and Watkins (1997, p. 478) also wrote about how ‘alliances can weaken as well as invigorate a movement’, and here we find their analysis prescient. The alliance with neo-Malthusians has, in many ways, weakened the feminist reproductive rights movement worldwide, even though it has helped that movement gain visibility, status, and funding.

ICPD+30, however, may serve as a moment of invigoration for the global feminist movement for reproductive rights and justice. This time of both retrospection and planning for the future presents an opportune moment to reflect critically on the alliances forged at Cairo and the extent to which carrying them forward will be helpful or unhelpful. As the Covid-19 pandemic has transformed the world, and alarmism about global population decline is growing to match alarmism about population growth, those of us envisaging the future of global family planning might find this an opportune moment to reflect on which bedmates we want fighting along with us in the movement for reproductive justice. The ICPD+30 moment allows us to reconsider the compromises made at Cairo on issues of poverty, environmental protection, racism, and coloniality and to create new ways of addressing these challenges without implicitly blaming poor women’s own bodies and their fertility. As we confront multiple interlocking crises, it is time to consider their structural root causes and how these structures may be radically transformed. Whom we are in bed with, then, as we confront these challenges becomes all the more important.

Compromise, of course, is not a bad thing, and it is indeed necessary for movement building and for social change. The reproductive justice movement, by definition, requires the building of bridges and forging of alliances across struggles to build power and to reflect the multidimensional challenges that people face. But reproductive justice also requires a brave imagination: to seek nothing short of true reproductive liberation and to stop settling for crumbs in the meantime. In this moment of ICPD +30, rather than seeking to protect the compromised vision in fear of a backlash or further backsliding, unveiling how these compromises came to be codified as the set standard can show a way forward for a transformative agenda. Mainstream feminists in the Global North must hop out of their proverbial bed with the population controllers to forge new and stronger alliances with feminists in the Global South (Mohanty et al. 1991). These groups can reclaim the revolutionary feminist vision of reproductive rights from the population controllers and revoke the pass the latter has had since the ICPD to shroud neo-Malthusian family planning programmes under the banner of women’s health and empowerment.

## Figures and Tables

**Table 1:** Aims of organizations working on SRHR and family planning

International Women’s Health Coalition (IWHC) (2020) ^1^	Our Vision: A just and sustainable* world where all people, regardless of gender, enjoy their human rights and health, and have power over their lives. *A world that balances economic, environmental, and social dimensions to ensure every person is able to enjoy their human rights and dignity equitably without compromising quality and availability of finite resources in the future.
Population Council (2024)	Our Mission: We generate ideas, produce evidence, and design solutions to improve the lives of underserved populations around the world. We take a multidisciplinary, intergenerational, life-cycle approach that contributes to four global goals, including the connections between them:
	1. Ensure sexual and reproductive health, rights, and choices 2. Empower adolescents and young people to reach their full potential 3. Achieve gender equality and equity 4. Pursue justice in the face of climate and environmental changes
International Planned Parenthood Federation (2022)	Our strategy responds to social, political, and demographic global trends. These include: the expectations and potential of the largest ever generation of young people; ongoing, significant social and economic inequalities, including discrimination against girls and women; and opposition that threatens gains in human rights.
Population Services International (PSI) (2020)	PSI’s role as a leading global supplier of modern contraception and family planning/reproductive health programmes contributes to the environmental sustainability of the countries where we work. Helping to meet the unmet need on the part of the 230 million women for modern contraception is fundamental to the rights of women, as well as the sustainability of their communities.

1 In June 2021, IWHC merged with the Center for Health and Global Equity and IPPF Western Hemisphere Region, relaunching in October 2021 as Fòs Feminista.

*Note*: All wording in the second column is direct quotes from the organizations, but the underlining for emphasis is ours.
